# Technology in scientific practice: how H. J. Muller used the fruit fly to investigate the X-ray machine

**DOI:** 10.1007/s40656-023-00572-9

**Published:** 2023-06-02

**Authors:** Svit Komel

**Affiliations:** University of Ljubljana, Ljubljana, Slovenia

**Keywords:** Technology, Sedimentation, Model organisms, Instruments, Hermann J. Muller

## Abstract

Since the practice turn, the role technologies play in the production of scientific knowledge has become a prominent topic in science studies. Much existing scholarship, however, either limits technology to merely mechanical instrumentation or uses the term for a wide variety of items. This article argues that technologies in scientific practice can be understood as a result of past scientific knowledge becoming sedimented in materials, like model organisms, synthetic reagents or mechanical instruments, through the routine use of these materials in subsequent research practice. The proposed theoretical interpretation of technology is examined through a case where a model organism—*Drosophila melanogaster*—acted as a technology for investigating a contested biological effect of a mechanical instrument: Hermann J. Muller’s experiments on X-ray mutagenicity in the 1920s. The article reconstructs how Muller employed two synthetic *Drosophila* stocks as tests for measuring X-rays’ capacity to cause genetic aberration. It argues that past scientific knowledge sedimented in the *Drosophila* stocks influenced Muller’s perception of X-ray-induced mutation. It further describes how Muller’s concept of X-ray mutagenicity sedimented through the adoption of X-ray machines as a ready-made resource for producing mutants by other geneticists, for instance George Beadle and Edward Tatum in their experiments on *Neurospora crassa*, despite ongoing disputes surrounding Muller’s conclusions. Technological sedimentation is proposed as a potential explanation why sedimentation and disputation may often coexist in the history of science.

## Introduction

Since at least Bachelard (1938, 1951) and Heidegger (1977), it has been repeatedly asserted that science’s reliance on technology distinguishes it from other forms of knowledge. In recent decades, exploring the impact of technology on scientific work has become somewhat of a trademark among authors associated with the so-called practice turn. Notwithstanding their numerous rewarding insights, these accounts have based their notion of technology mostly on mechanical instruments. Even when the focus shifted to non-physical sciences, the archetypes of technology remained pieces of apparatus, like spectrometers, microscopes, lasers, ultracentrifuges, etc. (Hacking, 1983, 1992; Collins, 1974, 1985; Lynch, 1985; Galison, 1987; Lenoir, 1986; Baird, 2004; Gooding et al., 1989; Pickering, 1995) On the other hand, in attempts to capture a more extensive gamut of implements, the terms “technologies” and “tools” tend to cover a broad array of items. One influential example of a wider understanding of technology is Steven Shapin and Simon Schaffer’s tripartite scheme of material, social and literary technologies (Schaffer, 1988, 1992; Shapin, 1984; Shapin & Schaffer, 2011[1985]).^1^ Treating rhetorical devices, pictures, organisation of space, discipline and training as technologies provides an innovative method for highlighting their role in the construction of scientific knowledge. However, the only characteristic that these strategies and objects have common, according to Shapin and Schaffer, is being means for producing facts and making these facts appear as objectively given, rather than manmade—a trait attributable to most elements of scientific practice. Since they essentially equate technology with manner of knowledge production, their interpretation makes it difficult to distinguish technology from other facets of research. Whereas Shapin and Schaffer identify an important common feature of technology—the creation of seemingly objective matters of fact—later comparable accounts tend to be vaguer and do not address at all what constitutes something as a technology in scientific practice. One STS-inspired volume, for instance, devoted to the role of tools in the life sciences, employs this notion as a metaphorical umbrella term encompassing everything from screwdrivers, saws, instruments and organisms to statistics, inscriptions, mathematics, concepts and even entire disciplines (Clarke & Fujimura, 1992, especially the introduction and contributions of Griesemer, 1992, and Holmes, 1992; see also Feest, 2010).

In contrast, I argue for a notion of research technology both broader and narrower than these approaches, which goes beyond equating technology with mechanical artefacts, while aiming to specify its unique nature among other elements of scientific knowledge production. I propose that technology in research practice can be considered as an outcome of past scientific knowledge becoming sedimented in materials that are employed routinely in new investigations of puzzling phenomena. The term *sedimentation* is borrowed from Husserl, who used it to describe the process of established scientific concepts transforming into passively received thought. Through sedimentation, concepts that were once actively examined and contested become taken for granted. As will be shown, sedimentation occurs when concepts are shared through a perceptible medium, though Husserl mostly limits himself to language (Husserl, 1970b; Merleau-Ponty, 2002). Expanding on his remarks, I argue that technologies result from scientific knowledge becoming sedimented in particular material shapes which can be used to physically interact with investigated phenomena. Following this definition, instruments represent merely one kind of technology alongside standard laboratory organisms, synthetic reagents and other materials with which scientists may probe their research objects. These particular types of technologies can be distinguished from one another according to the different knowledge sedimented in them and their unique physical properties which allow distinct ways of manipulating research phenomena.

The article develops this interpretation of technology by examining a historical episode in which an organism—the fruit fly (*Drosophila melanogaster*)—served as a technology for investigating a poorly understood biological effect of a mechanically produced physical phenomenon—the mutagenic effects of X-rays—in Herman J. Muller’s experiments in the 1920s. Until 1915 Muller was one of the leading workers in Thomas Hunt Morgan’s famous Fly Room at Columbia University where *Drosophila* was first developed into a technology for genetic analysis. After leaving Columbia, Muller began to use specimens of fly mutants to determine the effect of temperature and radiation on the frequency of mutation. In the most successful of these experiments, for which he received the Nobel Prize in 1946, Muller relied on specific *Drosophila* strains as tests for measuring the mutagenicity of Roentgen radiation.

In Sect. 2, I outline the general features of technological sedimentation and compare my view to other authors who have similarly suggested that technologies can be regarded as reified scientific thought. Section 3 recounts how *Drosophila* mutants were constructed into technologies for genetic research, focusing especially on the particular concepts of the gene and mutation that sedimented in the flies and impacted Muller’s later X-ray experiments. Section 4 details how Muller exploited two synthetically designed fly stocks to measure the mutagenic effects of X-rays. I argue that due to the genetic knowledge sedimented in the fly stocks, the *Drosophila* technologies influenced how Muller framed and perceived the problem of X-ray mutagenicity. Furthermore, I show that Muller attempted to abstract from the results he obtained with the flies a much more general conclusion, alleging that X-ray-induced mutations resembled naturally occurring mutations. By considering the claims Muller made after performing his experiments, I demonstrate how technologies may generate underdetermination. In Sect. 5, I contend that Muller’s concept of X-ray mutagenicity sedimented by virtue of other geneticists adopting the X-ray machine as a ready-made resource for inducing mutation in various organisms. In parallel, some researchers still scrutinized Muller’s interpretation of X-ray mutagenicity. It follows that technological sedimentation does not presume a complete resolution of disputes, but can rather help explain why users of the X-ray machine took Muller’s concept of X-ray mutagenicity for granted despite new findings raising potential doubts against Muller’s initial claims.

## Technological sedimentation

Treating technologies as stores of reified past scientific knowledge finds support in remarks made by several previous writers (e.g., Heidegger, 1977; Fleck, 1986[1947]; Wise, 1993). Two authors in particular offer more elaborate accounts which inform my position: Hans-Jörg Rheinberger and Bruno Latour.

In his historical analyses of post-war research on protein synthesis, Rheinberger (1992a, 1992b, 1995, 1997) introduced a distinction between technological and epistemic things. With the latter he denotes material entities that constitute the object of inquiry in a given experimental system. Epistemic things represent a difference, a surprising result, at which practitioners direct their experiments without yet having a clear idea with what exactly they are working. Once understood and established, epistemic things transform into technologies, which for Rheinberger comprise instruments, organisms, reagents, kits and other materials that form the stable basis of an experimental system. One example are radioactive amino acids, employed as tracer molecules for investigating biosynthetic pathways. Technological objects thus “embody the heavy load of knowledge taken for granted at a particular time” (Rheinberger, 1997). Having become settled as “reified theorems”, they are handled routinely and usually outside the line of research in which they initially emerged as epistemic things. What constitutes an epistemic or technological thing at any given time depends on its application in a particular research setting. No object is categorised eternally or in itself. Rheinberger’s analytical framework is attractive, in my opinion, precisely because it captures this fluid interplay between science and technology in experimental practice, instead of reducing it to a distinction between basic and applied research or science versus engineering. However, Rheinberger does not focus on the technological side, but is rather concentrated on scientific activity as a “generator of surprises” (1997). Although he lays the groundwork for a theory of technology, he pays less attention to the transformation of research objects into devices than to how experimental research transcends its stable technological conditions by creatively tinkering with unanticipated phenomena.

Latour paints a cognate picture of technology in his earlier individual work (1987) and collaboration with Woolgar (1986). Like Rheinberger he is inspired by Bachelard and regards pieces of apparatus as reified theory. Also related is Latour’s point that skills, procedures, instruments and documents embody the end-results of controversies in a given field. Once arguments are resolved, knowledge produced within a discipline is packaged into *black boxes*, devices that may be rallied outside their initial setting, in other laboratories, where they function as a foundation for future research (Latour, 1987).^2^ The mass-spectrometer, say, embodies conceptual contents of physics. In sum, the technological equipment of each laboratory “represents the reification of knowledge established in another field” (Latour & Woolgar, 1986). While Latour and Woolgar’s account of black boxes is to an extent compatible with my view, I have several issues with their approach to technology. First, most of their examples are mechanical instruments, like the mass-spectrometer or centrifuge. Second, when they move beyond black boxes of physics, they tend to group together an unselective bundle of items, which among other things also include routinized technical procedures, such as bioassays or chromatography (Latour & Woolgar, 1986). In my opinion these are better understood as *techniques*, i.e., sequences of standardised practical operations, which are distinct from reified, material technology (although the two depend on one another, as I explain in the continuation of the paper). Finally, aside from the alternating mechano-centrism and vagueness, Latour and Woolgar overhastily adopt the practitioners’ perspective that literary output is the *raison d’être* of laboratory activity. This tendency is reflected in the notion of *inscription devices*, according to which the essential common characteristic of technologies is that they produce inscriptions—figures, diagrams, graphs—which are directly published in scientific texts and used as arguments in disputes (Latour & Woolgar, 1986). In other words, what supposedly justifies equating diverse phenomena like statistics, programming languages, machines and technical skill as devices is their production of documents that can be mobilised to resolve controversies and create facts (Latour, 1987; Latour & Woolgar, 1986). Material technology and technical practices are thus subordinated to writing and visual imagery, regarded as the main goals of laboratory work.^3^ This version of Latour has been relatively widely emulated in science studies, not least by Rheinberger who also attaches primary importance to the “signifiers of science” or what he calls “graphematic traces”—charts, micrographs, ultracentrifugal patterns, etc. (Rheinberger, 1992b, 1995, 1997).

Neither Rheinberger nor Latour devote much attention to how past scientific results actually become reified in technologies. Both mostly limit themselves to observing that the transformation of successful, undisputed scientific knowledge into technologies is a constantly occurring process, and instead focus on how technologies shape research phenomena into “bundles of inscriptions”, to borrow Rheinberger’s (1997) Latourian expression. My aim is to reinterpret this reification of past scientific knowledge as a process of sedimentation, which I believe to be constitutive of technologies in scientific practice, and analyse how the circumstance that technologies are results of sedimentation affects subsequent research performed with them.

Husserl (1970b) introduced the term sedimentation in his *Origin of Geometry* (written 1936, published 1939) to describe a socio-historical process through which scientific concepts turn from an object of investigation and debate into taken for granted, ready-made concepts that scientists apply routinely. Sedimentation implies a passive adoption of past concepts as acquired tradition, without consciously reflecting on the origins of their meaning. This is why Husserl also designates it as “traditionalization” of scientific knowledge. Sedimented concepts are employed unthinkingly, without retracing the activities that had initially given rise to them. The history of “the whole toilsome work of achieving” these concepts is hence forgotten and “takes on the character of a mere pathway to a goal” (Husserl, 1970a). In science, Husserl explains, some sedimentation is unavoidable as it allows each worker to focus on their part of the building, without having to run “through the whole chain of groundings back to the original premises” (1970b). New results are attained based on past acquisitions and in turn become working materials for other findings. Scientific thought is thus continuously realised through sediments of forgotten former activity, which provide a foundation for producing new knowledge (Merleau-Ponty, 2002). Moreover, sedimentation is not only imperative for the progression of thought, but represents for Husserl the manner in which concepts may be shared beyond the level of the individual subject as ideas held in common—as “social knowledge” or “general intellect”, one might say, even though Husserl and his interpreters would avoid those terms (Buckley, 1992; Merleau-Ponty, 2002).

Sedimentation thus occurs when knowledge is shared and becomes a thing in communal use. But to be made accessible to others and shareable, thought must be embodied in a perceptible medium. In *Origin,* Husserl mentions merely linguistic modes of expression—speech and especially writing. In this article, however, I would like to highlight a particular mode of sedimentation that occurs when scientific thought sediments in instruments, organisms, reagents, kits and other materials used in scientific practice, by virtue of which these materials come to act as technologies in scientific research.^4^ As outcomes of this distinct form of sedimentation, technologies possess a dual quality: (1) a concrete, physical existence as a material thing, and (2) an abstract quality as a sedimentation of mental products of past scientific work. Both the physical and the conceptual quality are significant. The material form of technology allows concepts, sedimented in it, to be brought upon nature. Whereas linguistic or visual forms lack the appropriate shape to physically interact with phenomena emerging in scientific practice, technologies reintroduce past sedimented concepts into current research in a material form that can be used to manipulate new objects of investigation. Muller’s flies, for instance, yielded results about X-ray mutagenicity that were published in journals, newspapers, textbooks, presented at conferences and, eventually, taught in schools. But his concept of X-ray mutagenicity could not be applied as a mutant-producing technology in other laboratories had it not sedimented in the X-ray machine, which became a routinely used material in subsequent genetic experiments. On the other hand, because technologies are freighted with conceptual sediments, interventions with them are not neutral. They impose upon new objects a set of what I call *technological parameters*. These parameters are certain select characteristics that technologies isolate in the investigated phenomenon, which serve as handholds that researchers use to manipulate and understand the phenomenon. The particular characteristics by means of which scientists initially grasp unexpected phenomena therefore depend on the particular technologies with which they handle them.^5^

Although my analysis of technological sedimentation is inspired by Husserl’s term, I will depart from his interpretation in several interrelated aspects. First, I agree with Husserl that the object of sedimentation are concepts and the accompanying propositions which fix their meaning and relation to other concepts. What sediments are thus not full-fledged theories, but rather more circumscribed abstract descriptions of a phenomenon, property, process or reaction, like X-ray mutagenicity, crossing-over, gene-enzyme relationship, etc. However, Husserl overlooked that the sedimentation of these abstractions occurs through their practical use. When we read an article or listen to a lecture about the gene, for instance, we do not merely receive a concept but also concrete examples of how this term should be applied in written or oral utterances. By experiencing socially situated instances of communication, we spontaneously also learn conventions of using received concepts; to the extent that most scientists and lay-people can adopt the term gene in their own everyday use without necessarily meditating on the precise meaning ascribed to the word. Similarly, past scientific concepts associated with research materials like the fruit fly or X-ray tube become sedimented knowledge for practitioners as they use these materials in concrete experimental situations. The more they work with these materials, the more practical situations they experience, the more routine becomes the use of these technologies and the more taken for granted, familiar and inconspicuous become the conceptual abstractions sedimented in them. Much like one does not need to know precisely what a gene is to form meaningful utterances with the word, merely be conversant with the rules of using it in a sentence, it suffices for a biologist to be capable of operating an X-ray tube (or recognising when to have someone operate it) to conduct experiments on genetic mutation, without scrutinizing the established scientific knowledge about X-rays.

Second, the practical nature of sedimentation is why I propose technologies be distinguished from *techniques*—sequences of practical operations for working *with* technologies in experimental situations (cf. Jordan & Lynch, 1992; Latour, 1994; Rapp, 1981). Whereas the concepts sedimented in technology usually represent explicit, theoretical knowledge, techniques comprise manual and perceptual skills that presuppose rehearsal and bodily discipline. The two types of knowledge are obviously mutually dependent: practical expertise can often be theoretically codified and operating a technology inevitably demands an appropriate assortment of skills (Collins, 1990; MacKenzie, 1996). But sedimentation can occur precisely because the concepts sedimented in technologies and the techniques for using them may exist and develop separately. One can master the use of a technology while taking for granted the concepts sedimented in it. Within each historical site of scientific production, we can thus identify a particular combination of technologies and techniques that drive them. When concepts sediment in new materials that are adopted in research, these new technologies typically bring about a reorganisation of the labour process and development of different techniques.

The practical nature of sedimentation is also closely related to my third point: both a medium and its use by a community of people are necessary for sedimentation to happen. Husserl mostly emphasises that sedimentation occurs by virtue of concepts becoming embodied in perceptible media. But it is just as important that these media are used by other people than their original creators. If no one adopts an expression introduced in an article, if scientists do not apply a material in their research practice, no sedimentation takes place. Sedimentation therefore presumes a community for which the sedimented concepts come to represent a shared tradition. At the same time, Husserl is right in emphasising that the form in which concepts are shared is not a neutral means of transmission.^6^ Because concepts are sedimented in a material like the fruit fly or the X-ray machine, scientists may use them in their practice without actively examining the concepts sedimented in these technologies. The medium enables techniques and sedimented knowledge to exist and evolve separately. Practitioners may master using a material in concrete situations without reflecting on how “they”, as a community of people who deploy this material as a ready-made thing, have come to know what this material is or how it works. By adopting the medium in this matter-of-factly manner, however, users also more-or-less tacitly accept the conceptual abstractions sedimented in it. The medium therefore facilitates the undeliberated way of receiving past scientific concepts, which is characteristic of sedimentation.

Finally, I distance myself from Husserl’s view of sedimentation as a unidirectional process, a “continuous synthesis in which all acquisitions maintain their validity” (1970b; see also Hacking, 2010). Just as schematic, albeit less teleological, is Latour and Rheinberger’s shared belief that the transformation of facts into technologies only happens after these facts become undisputed (Latour, 1987; Latour & Woolgar, 1986; Rheinberger, 1997). As I will demonstrate in Sect. 5, sedimentation usually unfolds in parallel with ongoing disputes, even in textbook success stories like Muller’s. Technological sedimentation is not inhibited by ongoing controversy, but can rather help explain ambivalent cases in history of science where a community of researchers adopts a technology despite other scientists questioning the knowledge sedimented in it.

## Technologizing *Drosophila*

Before looking at Muller’s experiments on mutation frequency, it is imperative to recount how *Drosophila* first came to function as a genetic technology in Thomas Hunt Morgan’s laboratory at Columbia University. It is a well-known story how Morgan spotted the white-eyed fly mutant in 1910 and proceeded to recruit Calvin Bridges, Alfred Sturtevant and Muller, three students at Columbia, to work in his “Fly Room” (Sturtevant, 2001[1965]; Allen, 1975, 1978; Carlson, 1971, 1974; Kohler, 1993, 1994; Waters, 2004, 2008). The group of drosophilists epitomised in this elite quadruplet collaborated until 1915 when Muller left Columbia. The aim of this section is not to tell the story of the Fly Room anew, but to retrace some of the concepts sedimented in the fly stocks that prompted Muller to approach X-ray mutagenicity differently than contemporary scientists working with other organisms. In particular, I contend that the Morgan group’s tendency to treat fly mutants as embodiments of gene mutations led Muller to restrict the possible genetic effects of X-rays that were being studied to changes in individual genes.

When *Drosophila* first entered the laboratory, its chromosomes were filled with pre-existing mutations that randomly expressed themselves and flouted theoretical predictions. Since Morgan’s group worked in an era before synthetic or even molecular biology, the only available technique for modifying hereditary material were selective breeding procedures (Rheinberger & Gaudillière, 2004). By executing complex crosses, they were able to break down the genetic melange that had accumulated in the flies over their evolutionary history and obtain purified stocks. Through artificial selection and inbreeding, they gradually sequestered a collection of “good mutants” whose particular traits enabled them to serve as reagents for analysing particular hereditary processes (Carlson, 1974; Kohler, 1994; Waters, 2008). These useful aberrations could then be further combined into synthetically designed compound stocks, which amalgamated several known mutations.

As opposed to frog muscle tissue in physiology (Holmes, 1993) or algae in photosynthesis research (Zallen, 1993), *Drosophila* was not just a more convenient experimental organism, through which investigated phenomena would reveal themselves more plainly. Its pragmatic properties, like its short reproduction rate, were merely one side of the story. What made *Drosophila* special was that each examined strain was a potential genetic device, which could be brought to bear upon future abnormalities. Already purified and clarified fly specimens were no longer mere research objects, but primarily acted as technologies for studying inheritance in other mutants (which might be transformed into still new devices). Every “discovered” abnormality could be employed to determine the basic genetic characteristics of aberrations that appeared later. Thus, each stock was, successively, a surprising phenomenon and a technology. As the repository of stocks grew, it provided an ever more refined and multi-purpose toolkit for testing diverse genetic phenomena.

**Fig. 1:**
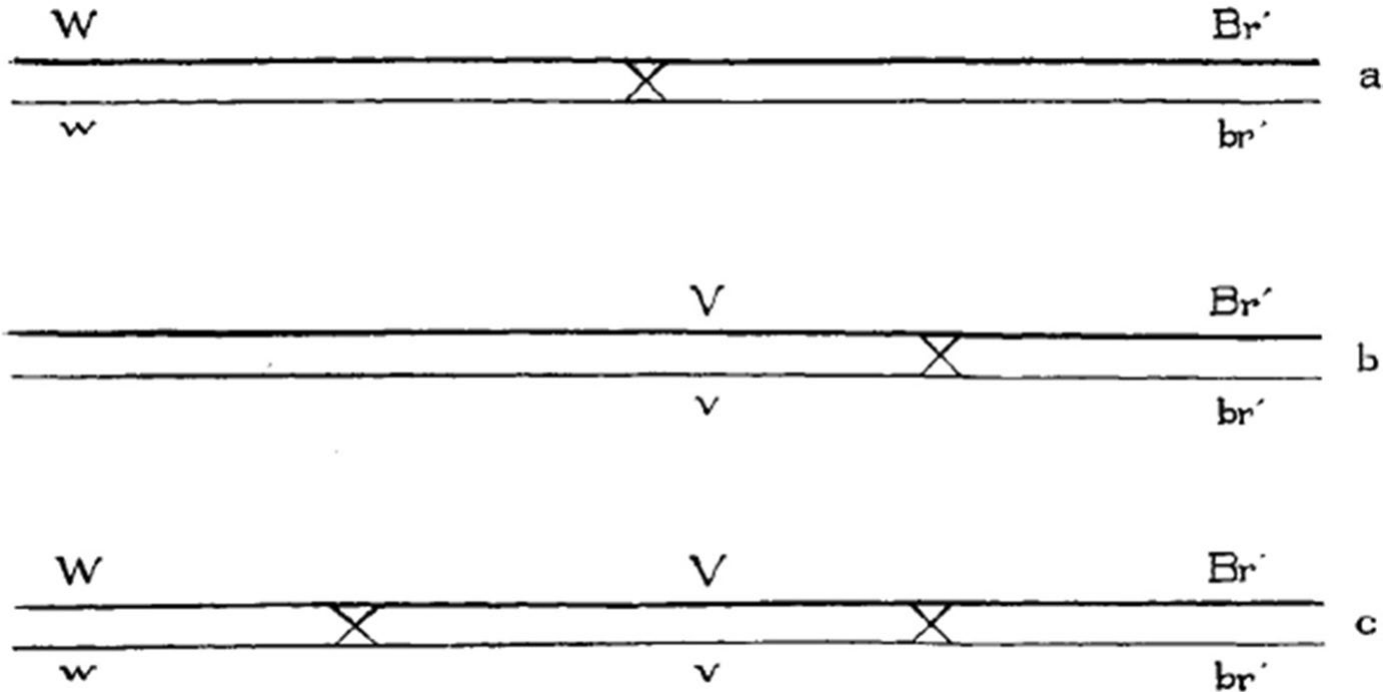
Linkage maps (redrawn from Tice, 1914). The maps depict the relative loci of white-eye (*w*), vermilion-eye (*v*) and bar-eye (*Br*) mutations on the X chromosome. Crossing-over is indicated by “X” between the lines, which depict homologous chromosomes

The three fundamental, interrelated concepts that sedimented through this work in all the flies, binding them together as specimens of the same technology, were linkage, crossing-over and the “factor” or gene as the basic unit of heredity. The general prediction, connecting these concepts, was that the closer two linked factors or genes are on the same chromosome, the lower the frequency of crossover events between them. Reduced to this parameter of recombination rate, all genes could be placed in a common virtual space: the linkage map (Fig. 1; Allen, 1978; Falk, 2004; Kohler, 1994). Fly specimens exhibiting new mutations could be crossed with existing ones to estimate their relative place on a chromosome*.* Previously mapped mutations hence served as marker genes or “identifying factors”, as they were also called at the time (e.g., Muller, 1928a). For instance, when a bar-eyed mutant was observed in 1913, it was first mated with the wild-type to establish that it was dominant and linked to the X chromosome (the wild-type here denoting the standard non-mutant laboratory strain, not an actually wild fly). It was then crossed with two already studied mutations located on the X chromosome to calculate its relative position. After its basic genetic properties were defined, the bar-eyed mutant was stored as potentially “valuable for linkage experiments” (Tice, 1914). This type of investigative approach, which started with an observation of a potentially interesting new phenotype and ideally resulted in obtaining a stable stock for future experiments, characterised the work of Morgan’s group (Waters, 2004).

Through years of repeated experimental work with the flies, the factor or gene came to be regarded within Morgan’s group as a hypothetical location on the chromosome that influenced outwardly perceptible characteristics of the organism. As members of the group worked with the flies, treating them as embodiments of different mutant factors or genes, they began to take the concept of the gene for granted. Many of their articles, especially later ones, use the terms “factor” and “gene” synonymously without explaining their meaning (Muller, 1916, 1918; Muller & Altenburg, 1919; Sturtevant, 1917; Tice, 1914).^7^ Individual factors and fly cultures were completely conflated in signifiers like “white-eye”, “barred”, etc., which were concomitantly used to denote a mutant gene and the purified stock that supposedly personified this gene. Although referring to a merely hypothetical entity, the concept of the gene consequently became as obviously real and manipulatable as the flies in which it had sedimented. A similar transformation happened with the corresponding concept of mutation. Through repeated use of the flies, the members of Morgan’s group came to assume that the perceived changes were predominantly due to *gene* mutations, not other forms of genetic aberration. Each fly stock was regarded as a ready-made embodiment of one or several “mutant factors” or “mutant genes” (Muller, 1916; Sturtevant, 1917). The linkage map is essentially a visual representation of this sedimented assumption, correlating points on individual chromosomes to stocks of mutant flies. Thus, as the concept of the gene sedimented in the flies, mutation also tended to be limited to changes in hypothetical segments of the chromosome. In the next section, it will be shown that this sedimented concept of mutation as gene mutation crucially affected how Muller framed X-ray-induced mutation as a research object.^8^

As I emphasised, sedimentation takes place through the practical use of materials in experimental situations. Making *Drosophila* mutants work as a technology therefore demanded the development of complex experimental techniques, as Waters (2004, 2008) has argued. When *Drosophila* had first entered the laboratory, it was considered a convenient practice material for students (Kohler, 1993). By the late 1910s, when the fly had turned into a sensitive genetic technology, commanding the several hundred existing stocks required an intricate know-how, from performing the breeding procedures (test-cross, two-point and three-point backcross, etc.) to preparing the food, maintaining a relatively constant temperature, etherizing, sorting and examining the flies, etc. Muller dismissed data from an experiment in 1918, which did not conform to his expectations, because it was carried out by students. The “labor of so many inexperienced persons”, as Muller (1928a) called it, so productively exploited in the early years, no longer sufficed.

Because genetic knowledge was sedimented in specimens of flies, it was possible to physically inflict it on other strains, either to produce synthetic stocks or to test new baffling mutations. Undoubtedly, the bar-eye and other mutations were presented in journals, textbooks and lectures. But without the vials of actively reproducing mutants as a material substrate, concepts distilled from the flies could not be employed in future research to extract additional knowledge. Making concepts operational crucially depends on an appropriate vehicle, which allows the intellectual products of past science to physically interact with other research objects. This material aspect is why I insist on distinguishing technology proper from what Shapin and Schaffer call literary technology, as well as Latour’s inscriptions. The most prominent inscription in Morgan’s lab was the aforementioned linkage map. Its purpose was to classify mutant genes, to order and compare data gleaned from years of crosses. However, without the flies the map would be like a treasure or a museum: a collection of well-organised valuable antiquities that lack the appropriate material form to twist nature into yielding new value. In order for past, sedimented knowledge to be mobilised in drawing out other unknown phenomena, it had to be invested in up-to-date fly mutants with the appropriate genetic traits for pinning down fresh research objects. As new concepts sedimented in the flies, they modified the material shape of the technology. The design of the stocks was constantly refurbished to reflect the current state of genetic expertise, meaning that some of the older *Drosophila* mutants had to be discarded to free up space for cutting edge models of fly technology (Kohler, 1993).

**Fig. 2:**
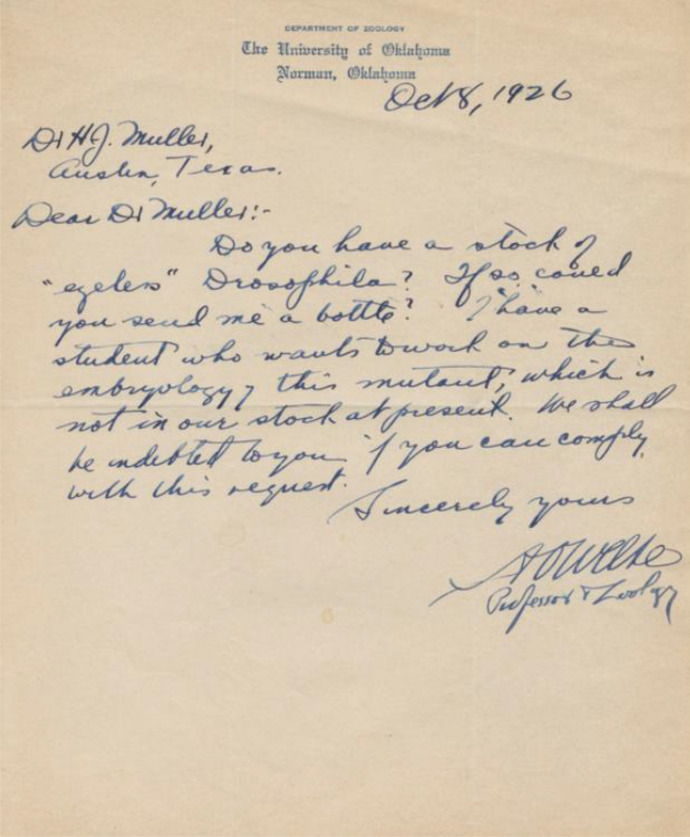
A letter from Asa Orrin Weese, professor of zoology at the University of Oklahoma, to H. J. Muller, asking for a bottle of eyeless Drosophila (1926). The body of the letter reads: “Dear Dr Muller! Do you have a stock of “eyeless” Drosophila? If so could you send me a bottle? I have a student who wants to work on the embryology of this mutant, which is not in our stock at present. We shall be indebted to you if you can comply with this request.” Courtesy Helen Muller and Lilly Library, Indiana University, Bloomington, Indiana. I sincerely thank Helen Muller for identifying the sender

Overall, though, the total amount of mutant commodities grew. In 1914 over a hundred strains were maintained at Columbia. Ten years later that number had more than tripled (Kohler, 1994). Practitioners recognised the paramount importance of their fly technology and created exchange networks for sharing specimens. Researchers visited distant *Drosophila* laboratories to obtain samples of state-of-the-art stocks or sent letters of requests to their colleagues (Fig. 2). Drosophilists also carried cultures of mutants with them as their prized possessions. Muller was perhaps the most extreme example of this *Drosophila* pilgrimage, migrating his fly fortune between the East Coast of the US and Texas, eventually shipping it all the way to the up-and-coming genetics facilities in the USSR. On 16 September 1933, when he took up a position at the Institute of Genetics in Leningrad, he brought with him “10,000 glass vials and 1,000 bottles” of *Drosophila* cultures (Carlson, 1981). In September 1937, while preparing for his departure from the Soviet Union, Muller made “subcultures of some 250 *Drosophila* stocks” before leaving for Paris (Schwartz, 2008). His expeditions give us an approximate geography of the Atlanticist research tradition that developed around *Drosophila* material culture. What defined and linked this tradition and its members was precisely a received, held in common, sedimented knowledge, bound with the stocks of fly mutants. And, vice versa, this knowledge acquired the status of a tradition as practicing drosophilists *traded* materials and expertise*.* Fruit flies only existed as technologies within this particular community, as embodiments of its traditionalized knowledge. If a mutant from a *Drosophila* laboratory had escaped into an adjacent physics department, it would have been regarded as an insignificant pest. At the same time, geographically dispersed researchers were tied into a community of drosophilists because they organised their practice around the fly and tacitly accepted it as an embodiment of common genetic knowledge. The process of sedimentation thus explains the intertwined meanings of *research tradition*, denoting both a habitual, common mode of acting and thinking whose historical origins are forgotten, as well as a community within which this received knowledge circulates and is recognised as shared culture.^9^

## Making X-ray mutagenicity fly

In 1915 Muller packed his fly cultures and left for Houston to take up a tenure at the recently established Rice Institute. He was joined there by Edgar Altenburg, his co-worker in the Fly Room. The geographical displacement came with a shift in research orientation. Within Morgan’s group the objective had been to identify the rare, spontaneously arising mutants and exploit them to decipher “normal” hereditary processes. The nature of mutations themselves, why they occurred and the various physical or chemical agents that potentially caused them remained elusive. After his departure from Columbia, these questions increasingly occupied Muller’s work. Individually and in collaboration with Altenburg, Muller used special *Drosophila* stocks as handles for approaching the problem of mutation. In doing so, he reduced X-ray-induced genetic change to a select set of parameters, which turned X-ray mutagenicity into a malleable object of investigation.

The main issue with experimenting on genetic variation was the diversity of circumstances that could influence it. It consequently seemed troublesome, if not impossible, to isolate the impact of a single agent. Aside from the plethora of substances to be tested, there existed a variety of possible genetic effects. These could range from gene or chromosomal mutation to other phenomena connected with inheritance—substances could alter chromosome reassortment or crossover frequency, cause non-disjunction, etc. Moreover, different criteria could be chosen as to which mutants should be counted (visible, lethal, recessive, dominant, sex-linked, autosome-linked). With “visibles”, i.e., mutations that affect outwardly noticeable properties, the observer’s perception may quickly compromise the count, considering that merely a few mutants would usually appear during experiments. Furthermore, it was questionable at which developmental stage mutagens acted, how they influenced viability and how to determine whether they arose in the experiment itself; all of which could distort the data. Finally, given the extremely low rate at which mutation occurred, it was hard to discern statistically significant effects from error. If Muller’s (1928a) count is to be trusted, the community of drosophilists found barely 400 mutants among approximately 20 million flies inspected between 1910 and 1926, a 1:50.000 ratio. Thus, even in experiments with thousands of specimens, a significant mutagenic effect of an agent would reflect itself in a minor difference of one or several mutants between the treated and control series, a divergence that could easily be criticised as artefactual noise.

When Muller turned to mutation studies, several attempts had already been made to “speed up” natural mutation by treating organisms with all kinds of substances. Soon after the discovery of X-rays in 1895, their influence on hereditary mechanisms was examined in multiple species, including frogs, higher plants and protozoa (Koernicke, 1904–1905; Bardeen, 1907). In 1907 Morgan and his student Fernandus Payne tried to provoke mutations in flies by subjecting them to heat, cold, centrifuging, X-rays, ultraviolet light, low pressure and other agents (Allen, 1975; Carlson, 1981). Morgan also assayed the effects of radium, acids, bases, salts, sugars and alcohol (Allen, 1975; Kohler, 1993). Daniel MacDougal, Charles Gager and Albert Blakeslee all tested radium in their respective experiments on plant genetics (Campos, 2015). By the summer of 1917, Morgan’s former student Harold Plough had surveyed the effects of both temperature and radium on the frequency of crossing-over in *Drosophila* (Plough, 1917, 1921, 1924). After Muller had started his first mutagenicity experiments, but before he moved to radiation, the radiologist James Mavor (1923) reported an influence of X-rays on crossover frequency and non-disjunction in *Drosophila*.

Muller therefore did not come up with the idea of artificially inducing genetic change nor with X-rays as the means of choice. His tactic was rather to reframe the mutation problem in a manner that allowed him to discredit past research as necessarily inconclusive, as Luis Campos (2015) has noted. Contrary to Campos, however, I do not believe that Muller's reassessment of mutation was primarily a theoretical choice fuelled by his metaphysical beliefs. It was largely influenced by the concepts sedimented in the flies through years of laboratory work. Between 1918 and 1920 Muller managed to compound some of the existing *Drosophila* stocks into “special genetic devices”, as he called them (1928a). The known hereditary properties of these strains allowed them to function as reliable detectors for particular types of mutation, which Muller adopted as parameters for measuring X-ray mutagenicity. In the remainder of this section, I describe how the fly technologies influenced Muller’s perception of X-ray-induced mutation. I will limit myself to his two most prominent devices: the *ClB* and the “sex-linked identifying genes” (SLIG) stocks (Muller, 1928a).^10^

**Fig. 3:**
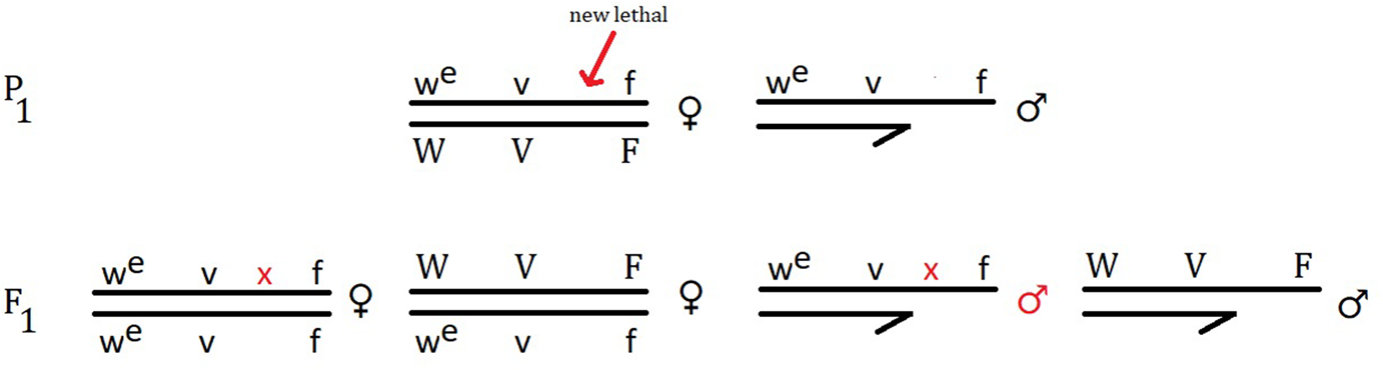
Sex-linked identifying genes test for detecting lethals (based on descriptions in Muller & Altenburg, 1919; Muller, 1928a). The “x” marks the new recessive lethal mutation. One F_1_ male is killed by the mutation arising on the X chromosome inherited from his mother. Lower case letters represent recessive alleles, upper case dominant ones. The marker genes are: eosin eye (w^e^), vermilion eye (v), forked bristles (f)

The SLIG is an improved version of the sex-ratio test, which Altenburg adopted in his earliest 1918–1919 experiments on mutation rate (Muller & Altenburg, 1919).^11^ The sex-ratio test is employed for detecting X-linked recessive lethal mutations. It consists of crossing two normal-type flies. All sons inherit their only X chromosome from their mother. One maternal X is transferred to half of the sons, the other to the rest. A new lethal mutation that appears on one of the mother’s chromosomes therefore kills half of the sons. The daughters obtain another mutation-free X from their father which prevents the recessive lethal from expressing itself. Thus, if a recessive X-linked lethal appears in a culture, it is revealed by a 2:1 sex-ratio, instead of the regular 1:1 proportion. When Muller and Altenburg met in the summer of 1919 at Woods Hole to repeat Altenburg’s earlier experiment, they did not cross wild-types but instead utilised a stock that Muller had refined for a similar experiment with his students in 1918. The females in this stock were heterozygous, containing three marker genes spread across their two Xs. The males carried matching recessive traits on their only X (Fig. 3). Each half of their sons would exhibit different characteristics, depending on which maternal X chromosome they inherited. Consequently, it was not merely possible to detect the presence of a lethal, but also determine on which maternal chromosome it arose by examining the markers in the surviving sons. The daughters carrying their mother’s lethal could also be distinguished more efficiently from their non-mutant sisters and reused in the next round of breeding (Muller & Altenburg, 1919; Muller, 1928a).

**Fig. 4:**
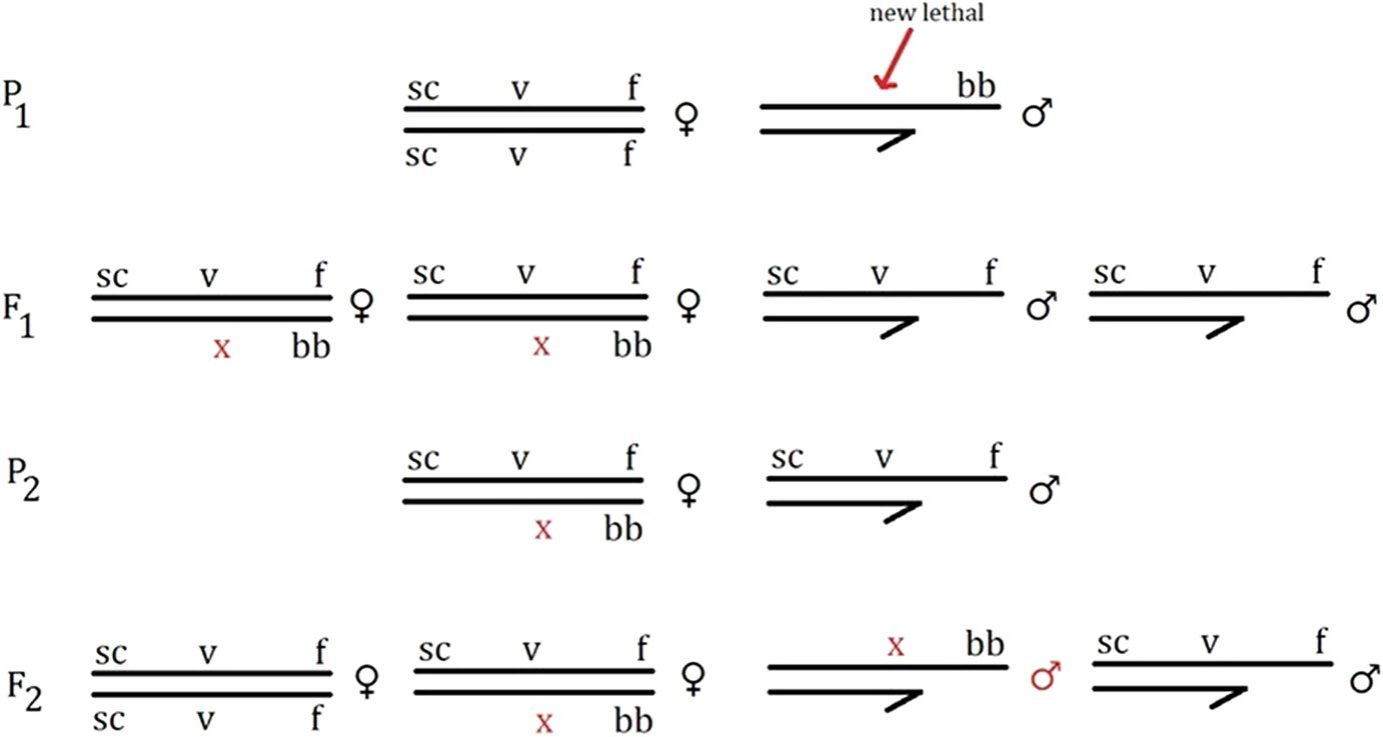
Test in Muller’s first X-ray experiment (autumn 1926) for screening recessive X-linked lethals in irradiated male gametes (based on Muller, 1928b). The scheme represents the scenario where mutation appears in the treated male flies. The “x” marks the recessive lethal mutation arising in the P_1_ sperm. This mutation expresses itself in the death of the F_2_ *bb* male. The markers are all recessive: scute bristles (sc), vermilion eye (v), forked bristles (f), bobbed bristles (bb)

Muller adopted a similar test system in his first X-ray experiment seven years later. This time, however, he screened both males and females. Additionally, he switched the markers: homozygous scute-vermilion-forked (*scvf*) females instead of *W*^*e*^*VF/w*^*e*^*vf*, and bobbed bristled (*bb*) males in lieu of *w*^*e*^*vf*. Because the males were marked differently, one could easily detect mutations appearing on the male X chromosome. The flies were divided between the control and treated series. The treated males were further split into four groups, each exposed to a different duration of radiation. Treated females were segregated into two groups, receiving two distinct spans of radiation. Muller’s colleague, radiologist Dalton Richardson, first irradiated the flies. The X-rayed flies were then mated to virgin, untreated flies of the opposite sex. Mutations in maternal Xs were revealed by roughly the same test as described above. Screening for new lethals in the paternal *bb* X required an extra step (Fig. 4). In the first generation, all sons would normally survive, inheriting one of their mother’s mutation-free *scvf* Xs. The daughters received one maternal X and their father’s *bb* X, containing the recessive lethal that had been potentially induced by radiation. In the second generation, these daughters were mated to their *scvf* brothers. If a lethal had generated in the father’s X, it killed all *bb* grandsons, sparing only the male progeny carrying *scvf* characters. If, on the contrary, the *scvf* males were missing, the mutation was attributed to the untreated maternal X chromosome. In this way, Muller could simultaneously compare the frequency of X-ray-induced mutation with the rate of spontaneous mutation in the control group (Muller, 1927, 1928b; Carlson, 1981).

**Fig. 5:**
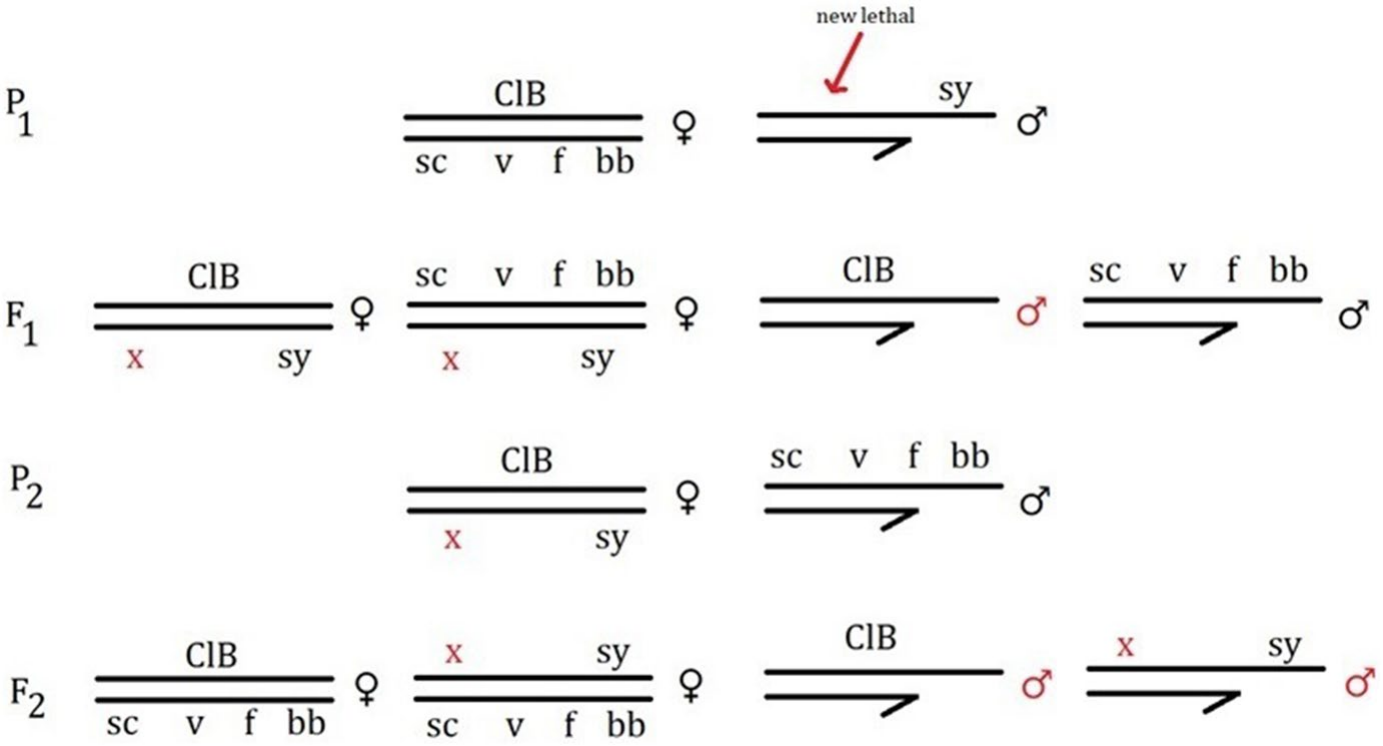
*ClB* test for screening recessive X-linked lethals in irradiated male gametes, used in Muller’s second X-ray experiment (based on Muller, 1928b). The *ClB* X chromosome also contained some other markers (sc, sm, v and t) which are omitted for sake of clarity, in accordance with Muller’s own notation. The “x” marks the recessive lethal mutation arising in the irradiated P_1_ father’s gametes. One class of males is killed by the *ClB* lethal, the other by the new X-ray-induced mutation on the paternal *sy* X. The marker on the sole male P_1_ X is small eye (sy). The markers on the second female P_1_ X are: scute bristles (sc), vermilion eye (v), forked bristles (f), bobbed bristles (bb). Lower case letters represent recessive alleles, upper case dominant ones

The second genetic device was the *ClB* stock. As opposed to the SLIG, it was not synthetically designed from existing strains, but emerged as a fortuitous accident in 1920. Muller, Altenburg and their flies met again at Woods Hole (Carlson, 1981; Schwartz, 2008). Muller was synthetising “an elaborate X-chromosome stock” in preparation for a much larger experiment than those conducted between 1918 and 1919 (Muller, 1928a). His ambitious design planned to compound more than a dozen markers, including the dominant bar-eye mutation. He failed, but in one of the last cultures he noticed a complete absence of bar-eyed male offspring. According to Altenburg, Muller instantly realised what had happened, exclaiming: “This is what I can use for lethals! It’s got a lethal in it and it suppresses crossing-over.” (cited in Carlson, 1981; Schwartz, 2008) Muller deduced that a mutant condition had cropped up on the female X chromosome, which concomitantly suppressed crossing-over and acted as a recessive lethal. Since it was composed of mutations previously encountered and explained in Morgan’s lab, it is not surprising that Muller immediately understood its “unexampled technical advantages” for detecting X-linked lethals (Muller, 1928a). Crossover suppression had been first encountered in a fly found in 1913 and analysed in detail by both Sturtevant and Muller (Muller, 1916; Sturtevant, 1917). The bar-eye mutant had already been explained and turned into a standard strain by 1914 (Tice, 1914). The new composite stock was labelled *ClB* (*C* for crossover suppression, *l* for lethal, *B* for bar-eye). It consisted of females who carried the *ClB* combination on one X chromosome, meaning that half of their sons would ordinarily die. If a new lethal emerged on the other X, it would kill the rest of the male progeny (Fig. 5). Appearance of an X-linked lethal was therefore reflected in a 1:0 sex-ratio, an even more absolute yardstick than the SLIG stock. Vials could be examined with the naked eye or lens without etherising the flies, thus minimising the risk of killing or sterilising them. Due to different markers, the daughters inheriting the *ClB* mutation could be effortlessly segregated from their sisters. The absence of crossing-over also kept the ratios of offspring more constant. Furthermore, because only one type of males could survive, the females did not have to be kept virgin, “a procedure that otherwise occupie[d] perhaps a third of the working time” (Muller, 1928a). Thanks to its beneficial traits, the *ClB* mutagenicity test is still regarded as Muller’s lasting technological contribution to genetics. A non-lethal variation of it remains in use today as a standard stock named “Muller-5” or “Basc” (Crow, 1987; Graf et al., 1992; “M5 technique”, 2006).

Muller’s two mutagenicity tests demonstrate how technologies impact the investigation of new phenomena. The SLIG and *ClB* stocks were material embodiments of sedimented concepts. The marker genes in these fly stocks, like bar-eye, all represented ready-made “mutant genes”, acquired from past *Drosophila* research. The concepts of crossover suppression, sex-linkage, zygosity and the gene had also become sedimented knowledge through repeated practical use of mutant stocks within Morgan’s group. Due to the particular concepts sedimented in them, the stocks imposed a set of parameters bringing out certain features of X-ray mutagenicity, while excluding other circumstances from consideration. As I indicated above, contemporary researchers suspected that X-rays affected hereditary material in various ways. Muller’s stocks reduced these manifold genetic effects to just four parameters: (1) recessive, (2) lethal, (3) X-linked and (4) gene mutation. All other types of abnormalities were eliminated from the count. Muller (1928b) explicitly acknowledged that his tests were incapable of detecting autosomal lethals. Occurrences in the Y chromosome were also neglected. Recessive (autosomal) visible mutations similarly evaded detection and became apparent only after several generations of crossings. Even if they were perceived, visibles not situated on the X chromosome were hard to identify, since no marker genes were inserted in the stocks’ autosomes. Despite not pursuing them systematically, Muller (1928b) took note of visibles that emerged, but did not add them to the final tallies which were compared in order to determine the frequency of X-ray-induced mutation in relation to the spontaneous mutation rate. Thus, the *Drosophila* technologies limited the diverse mutagenic effects that X-rays might provoke to a purified object consisting of merely four select parameters. They framed X-ray-induced mutation as a precisely defined type of change occurring in individual locations of the X chromosome. By excluding other abnormalities, the tests allowed Muller to detect and measure X-ray mutagenicity as variation in these parameters. The technological parameters therefore functioned as handholds, restricting a convoluted natural property to a simplified object, with which one could experiment in a controlled manner. Gripping nature by these parameters, Muller could disentangle a clear-cut influence of X-rays on the rate of mutation.

Having used the two fly stocks to reframe X-ray mutagenicity into an object consisting of the indicated four parameters, Muller could criticise the results of competing scientists who belonged to research traditions constituted around other organisms-technologies. First, Muller limited the diverse genetic aberrations, which could hypothetically be examined, to mutations in individual *genes* (Campos, 2015; Muller, 1928a). While Campos (2015) has already emphasised this point, he underestimates the extent to which Muller’s approach stemmed from the technologies he relied on. As I showed in the previous section, Morgan’s group came to assume that most fly strains were embodiments of one or several mutant genes. Given the *Drosophila* technology Muller was using, he was inclined to perceive the genetic changes happening in the stocks as gene mutations. The influence of the distinctive concept of mutation, sedimented in the fly, becomes particularly evident if we compare Muller’s view to geneticists radiating plant organisms. Blakeslee and Gager (1927), testing the influence of X-rays in Jimsonweed, distinguished between “chromosome and gene mutations”. Lewis Stadler (1928a, 1928b), working with maize and barley, spoke of the “genetic effects of X-rays”, which he understood to also encompass the influence of X-rays on the frequency of crossing over and chromosome deficiency. Past concepts, sedimented in the *Drosophila* mutants that Muller adopted in his practice, therefore compelled him to reduce the effects of X-rays to gene mutation. Second, Muller contested other researchers’ decision to choose visible mutants as indices of mutation, claiming that visibles represented only a small fraction of all mutation and that their determination was too dependent on each observer (Carlson, 1981; Muller, 1928a, 1928b, 1929). Indeed, there were several borderline cases where even trained drosophilists had trouble distinguishing mere variation, like an awkwardly folded wing, from a genuine mutation (Kohler, 1993). It is not without relevance that Altenburg suffered from severe myopia, Sturtevant was colour-blind and Muller was practically incapable of seeing on his right eye (Carlson, 1981). Recessive lethals had not been picked before because they were much harder to detect (Muller, 1928a). They could remain hidden for several generations and even when they were eventually expressed, they simply resulted in part of the offspring missing from the vials. Muller’s technologies, however, allowed him to spot this invisible absence, both due to their physical properties and the sedimented knowledge which limited possible interpretations of the genetic changes arising in the stocks. Third, Muller criticised the qualitative, fragmentary nature of previous observation, arguing that mutation should instead be investigated quantitatively, as a *rate* (Muller, 1928b). According to Muller’s new standards of proof, none of the existing experiments operated with a large enough sample to produce “meaningful” data (Muller, 1927, 1928a). Judging past experiments against these evidential standards, Muller concluded that they indicated nothing more than the fact that “mutations cannot be produced *en masse*” by the tested agents (Muller, 1928a; also Muller, 1927, 1928b). Again, Muller could impose such an exacting criterion of what constituted sufficient quantitative data because the material properties of the fly stocks allowed him to maintain a much larger number of irradiated individuals than competing experimenters working on mammals or higher plants. The absence of crossovers in the *ClB* stock also increased the proportion of flies that “gave evidence” (Muller, 1928a), enabling him to maintain smaller cultures. Muller could consequently keep the flies in vials, instead of milk bottles, further optimizing the use of available laboratory space and increasing the number of specimens that could be included in an experiment. Aside from the known material properties of the fly technologies, Muller could quantify the mutagenic effect of X-rays because of the parameters imposed by his technologies, which reduced potential genetic effects to a well-defined type of mutation that was less equivocal and consequently easier to count.

The technological parameters were thus indispensable for isolating a mutagenic effect of X-rays from among the complex of naturally occurring aberrations. Yet Muller relied on these parameters in order to derive a concept of X-ray mutagenicity that would extend beyond the constraints of his technologies. Although the restrictive technological parameters were needed to make the effect of X-rays conspicuous to scientific cognition and subjectable to quantitative measurement, Muller's underlying claim was that his selection of parameters was representative of all types of X-ray-induced mutation; that recessive-lethal-x-linked-gene mutation can stand for X-ray mutagenicity in general; and that, consequently, his particular choice of technology did not matter. His aim was to establish a conceptual description of nature as nature presumably is, independently of these technological interventions ever taking place. In fact, the further that Muller’s concept of X-ray mutagenicity could extend beyond the technological parameters, which had been imperative for arriving at his concept, and the more his technologies could be disregarded, the more far-reaching his discovery would become.

An obvious gap lay between the technological parameters and the concept that Muller aspired to abstract from them. The two tests allowed him to measure that X-linked recessive lethal mutation arose with a 0.083% frequency in the control group, as compared with 7.96% in the series exposed to 24 minutes of radiation and 12.15% in flies given 48 minutes of treatment (Muller, 1928b). The conceptualisation of this result could range from a strict interpretation—“X-rays heighten the rate of X-linked recessive lethal genes in *Drosophila* gametes”—, to the more general—“X-rays are mutagens”—, to the most unqualified abstraction, endorsed by Muller (1929) himself: “[M]utations in general bear all the earmarks of the X-ray mutations […] even if not all of them have actually been produced by radiation.”

A leap therefore occurred in the transition from X-ray-induced mutation as a puzzling natural phenomenon, to the technological parameters imposed by using the flies, to Muller’s concept of X-ray mutagenicity. The choice of technology depended on the studied natural phenomenon—the technology had to be at first glance physically, as well as conceptually, suitable for holding onto certain material properties of the investigated object. To measure X-ray-induced mutation, Muller had to use a living being with specific genetic attributes (physical quality), which were comprehended through the sedimented concepts of past research (conceptual quality). Irradiating his 1915 monograph or an uncharted or different organism, in which the same pieces of knowledge had not sedimented, would not work. However, technological parameters are underdetermined by nature. In Muller’s experiments, the parameters isolated only a particular mutagenic effect of X-rays. The traits of the two *Drosophila* stocks made them capable of detecting merely certain types of mutation. This reduction of natural events to a few select parameters is to an extent discretionary, because there is no necessary reason in nature itself for the scientific object to be framed exclusively in this manner. Now if the aim was to stubbornly stick to these parameters, their underdetermination would not have significant consequences. Yet, as in Muller’s case, the claim typically being made is that the technological parameters can be forgotten because they are representative of the natural phenomenon in general, even if technological interventions had not restricted it to these parameters. The problem of underdetermination comes into play when this leap occurs, from a technologically parametrized object to an abstract description of nature as nature supposedly is, regardless of the technologies used. Subsequent experiments may always call the assumption of the parameters’ representativity into question by furnishing new information. It might be revealed, for instance, that radiation has an incomparably strong effect on *Drosophila,* that X-rays can produce only some types of mutation or that the flies were killed by other X-ray-related causes than gene mutation. In Sect. 5, we will see that some of these objections were indeed raised and later proven against Muller's initial findings.

Muller put substantial effort into generalising the interpretation of his results beyond the technological parameters which had made them feasible. He repeatedly asserted in his articles and presentations that the parameters singled out by his testing devices can be taken as representative of artificially induced mutation in general. First, he maintained that lethals were an appropriate parameter because they did not differ “in their essential nature” from other types of mutation (Muller, 1928a). Lethals, he claimed, may therefore be “considered as random samples of ‘ordinary’ gene mutations, so far as the loci involved, and the mechanism […] of the mutations are concerned” (Muller, 1928b). Second, he guaranteed that while most detected X-ray-induced mutations were sex-linked there was “ample proof that mutations were occurring similarly throughout the chromatin” (Muller, 1927). Third, he held that his choice of *Drosophila* did not affect his results (Muller, 1929). In this respect, he profited from the contemporary popularity of radiation genetics and the findings of other groups working on a similar problem with different organisms (Stadler, 1928a, 1928b; Blakeslee & Gager, 1927; Whiting, 1928; Goodspeed & Olson, 1927; Goodspeed, 1929). As others have remarked (Carlson, 1981; Crow & Abrahamson, 1997), Muller attempted to secure priority for his discovery by publishing a four-page article without much data or descriptions of his experimental designs and methods (Muller, 1927). This manoeuvre initially provoked suspicion among other scientists, but he succeeded in appeasing most critiques by presenting a more substantiated paper two months later at the International Congress of Genetics (Muller, 1928b). It also helped that Muller’s closest rivals, especially Stadler (1928a), accepted his priority. Consequently, Muller could turn these competitors into confirmations. Fourth and most importantly, Muller alleged that artificially induced X-ray mutations were of the same kind as natural, spontaneous mutations. His principal argument was that many of the visible mutants, noticed in his experiments, were similar to those described during the past sixteen years of *Drosophila* research. Thus, rather amusingly, he relied on a class of mutants that was excluded from his parameters to fend off critics. To substantiate his claim of similarity between the natural and artificial, Muller (1928b) performed separate tests to determine that X-ray-induced visibles were allelomorphic to previously observed natural mutants (i.e., that their mutant genes lay on the same locus in the chromosome). Additional crosses were executed to check whether artificial mutants’ hereditary behaviour replicated that of their natural counterparts (Muller, 1928b, 1929). On a more metaphysical level, Muller suggested that X-rays were similar to evolution itself, by alluding that electrons, like evolution, strike the cells at random. Having made these extrapolations, Muller (1927) declared: “The changes produced by X-rays are of just the same kind as the ‘gene mutations’ which are obtained […] without such treatment, and which we believe furnish the building blocks of evolution.” Accordingly, the title of his 1927 article, in which he first announced his conclusions, was simply entitled *Artificial Transmutation of the Gene*.

The arguments Muller employed to generalise and entrench his concept of X-ray mutagenicity beyond the parameters of his fly technologies can be called *strategies of sedimentation*: rhetoric devices, visual representations, metaphors, arguments of priority, etc., that aim to persuade other scientists to assume the newly proposed conceptual abstraction as a legitimate description of nature in their own practice. The more successful these strategies are, the larger the community of scientists among which this new knowledge sediments. Muller managed to gain broad support for his interpretation of X-ray mutagenicity, leading to what Campos (2015 p. 226) has appropriately described as the “near-excision of decades of earlier work from the historical record”. This is precisely the result of sedimentation. Instead of being treated as an object of decades-long research, X-ray mutagenicity turned into a received ahistorical truth, on which new research could be based. As Muller’s concept sedimented, the distinction between what he had done with the flies and the interpretation he abstracted from his results, became blurred. Even recent historiographical studies, examining Muller’s experiments in detail, tend to be persuaded by Muller’s strategies of sedimentation.^12^ Schwartz (2008 pp. 240–241), for instance, simply adopts Muller’s voice as his own: “Man had for the first time willfully manipulated the genetic material.” Whereas Carlson (1981 p. 150) surmises that the abundant data, clever design of stocks and planned steps by themselves “dispelled the doubts and created a sensation”.

## Sedimentation and disputation

Though achieving remarkably wide acceptance, Muller’s results did not dispel all doubt. The sedimentation of Muller's concept of X-ray mutagenicity did not preclude the persistence of research that addressed open questions in Muller’s experiments. The purpose of this section is to explore why some practitioners could take Muller’s concept for granted while it was being questioned by other contemporary researchers—how can sedimentation and disputation co-exist? I believe this seemingly contradictory situation can be explained at least in part by the fact that the sedimentation of Muller's interpretation of X-ray mutagenicity hinged not mainly on discourse or explicit resolution of controversy, but mostly transpired tacitly through the dissemination and use of a commonly available instrument—the X-ray tube. The existence of ready-made X-ray tubes allowed practitioners to immediately adopt these machines in their everyday work to produce mutants, without necessarily paying attention to debates about the implications of Muller’s understanding of X-ray mutagenicity. As the tube came to be used routinely by a community of geneticists, Muller’s concept of X-rays as artificial transmuters of genes would sediment, irrespective of new findings made by scientists in other areas of research, including Muller himself, who continued to study X-ray mutagenicity. Technological sedimentation can therefore help explain why Muller’s concept of X-ray mutagenicity could remain taken for granted despite being questioned and corrected by subsequent research.^13^

Muller himself tried to encourage the sedimentation of his interpretation of X-ray mutagenicity by proposing to other workers in classical genetics to employ the Roentgen machine to create a “series of artificial races for use in the study of genetic […] phenomena” (Muller, 1927). He offered the “readily-obtainable X-ray” as a “handle” for producing and studying mutation (Muller, 1929). Muller presented the X-ray machine as a technology which could give rise to a new research tradition that he called the “physiology of mutation-production” (1929). The tube was therefore deployed to attract members to a potential scientific community, bound together by its acceptance of the X-ray as a standard technology for producing mutants and, implicitly, Muller’s concept of X-ray mutagenicity as sedimented knowledge.

**Fig. 6:**
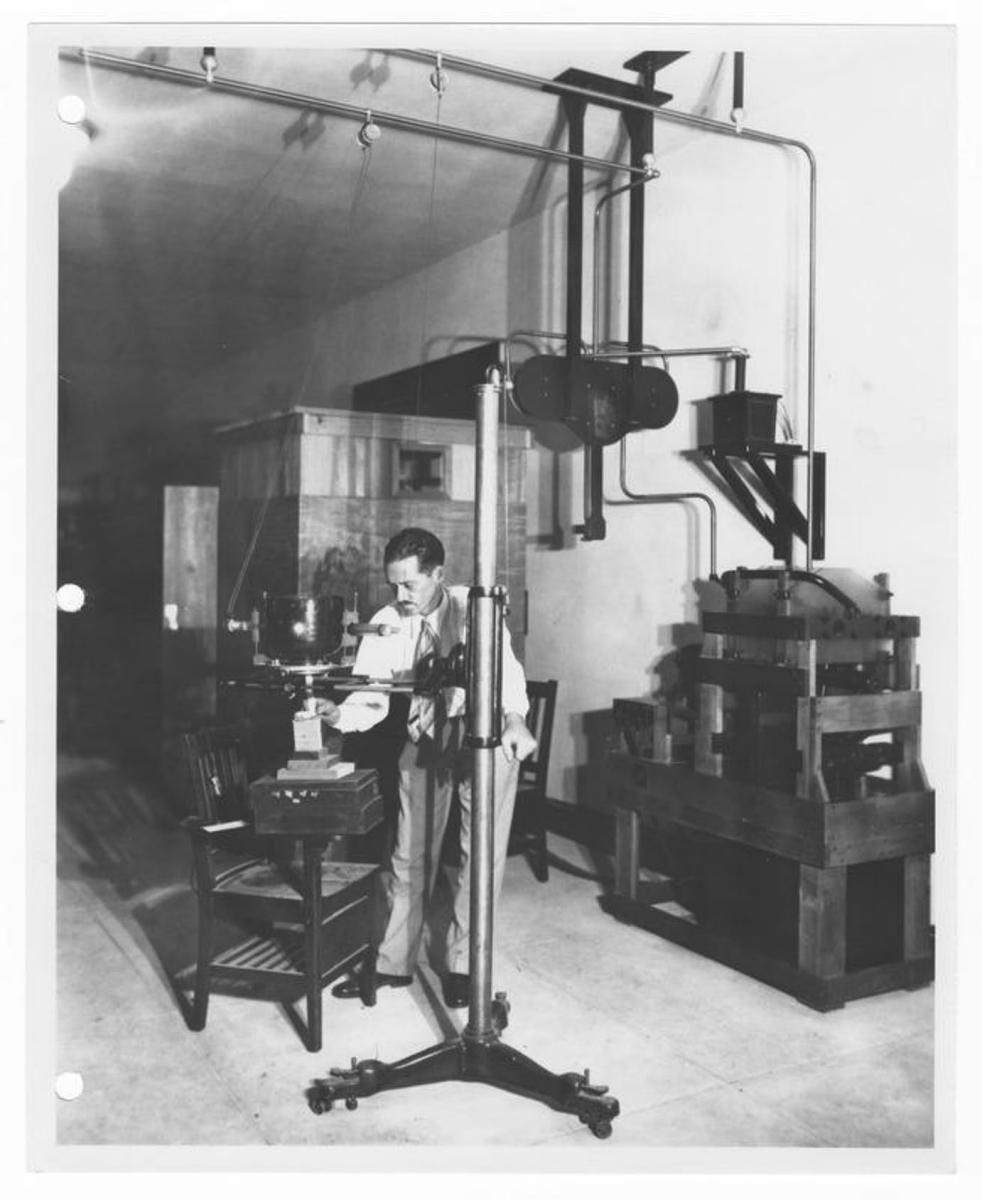
Muller’s graduate student, Clarence Paul “Pete” Oliver, working the X-ray machine at the University of Texas in 1927. Courtesy Helen Muller and Lilly Library, Indiana University, Bloomington, Indiana

**Fig. 7:**
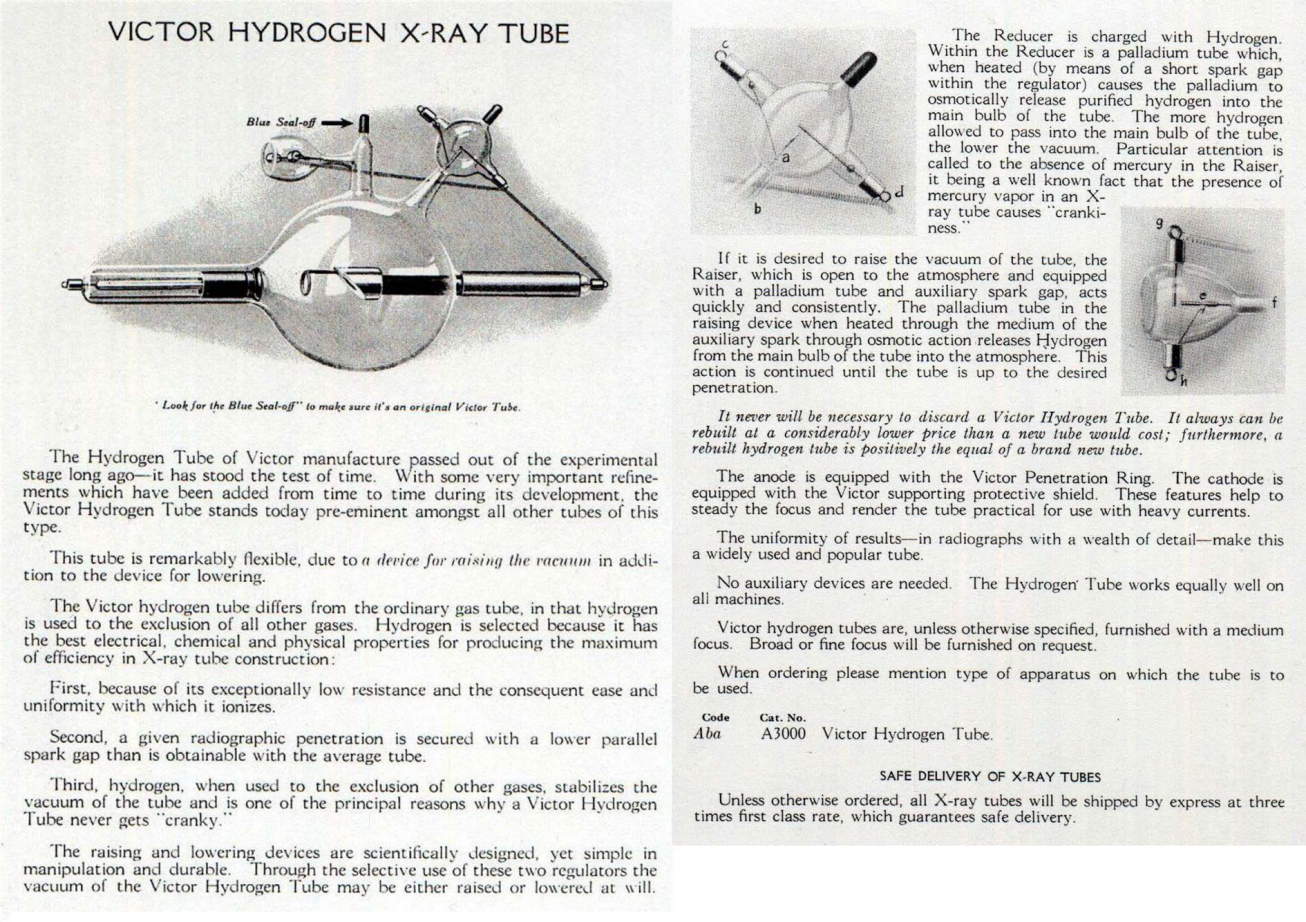
Victor catalogue description of the “Snook” hydrogen tube (Victor Corporation’s 1919 catalogue, pages 3–4, Medical Museion Collection, courtesy of Medical Museion, University of Copenhagen)

Many geneticists followed Muller’s proposal and adopted X-rays as “aids in experimental breeding” (Muller, 1928b). When Morgan’s group moved from Columbia to Caltech in 1928, it gained access to the powerful X-ray tubes designed by Charles Lauritsen in the adjacent nuclear physics department (Beadle, 1974; Carlson, 1981; Holbrow, 2003). Other genetics laboratories invested in their own X-ray equipment (Campos, 2015; Kohler, 1994). In this regard, a central factor contributing to the rapid sedimentation of Muller’s concept of X-ray mutagenicity was the commercial availability of Roentgen machines in the US. The machine that Muller borrowed for his 1926–27 X-ray experiments from the radiologist at the University of Texas was a “Snook” hydrogen tube, a catalogue model sold by the Victor X-ray Corporation. By late 1927, Muller’s laboratory had acquired its own tube, which was “of the same make” (Patterson & Muller, 1930; Fig. 6, 7). No further innovation was therefore necessary to craft a suitable frame in which Muller’s concept could travel to other scientific workstations and sediment. Some practitioners nevertheless modified the construction of the X-ray machine to enhance its gene-transmuting functions. One such custom-designed model was built in the 1930s at Stanford University by physicist Harry Clark and zoologist Morden Brown, experimenting on protozoa. Their apparatus had the same voltage as Muller’s but came with a modified metal construction, giving improved control over the intensity and constancy of the doses. It also added a more efficient cooling system, which was intended to minimise the potential impact of temperature on mutation (Taylor et al., 1933). Hence, in some cases, the sedimentation of Muller’s concept was reflected in a modification of the material shape of the X-ray machine. The second important circumstance, which facilitated the sedimentation of Muller’s concept of X-ray mutagenicity, was that a community of radiologists had already spread across US universities, supplying necessary know-how for operating X-ray machines. Their presence is recorded in numerous genetics articles, which acknowledge the help of local radiologists and physicists with conducting the experiments (Muller, 1927; Sax, 1938; Stadler, 1928a; Weinstein, 1928).

Muller’s concept was often assumed as a taken for granted basis for new experiments simply by virtue of X-ray tubes becoming used in everyday genetic research practice. A community of scientists developed, who applied the X-ray machine routinely, without thinking about how Muller had initially arrived at his interpretation of X-ray mutagenicity and without engaging in debates about X-ray mutagenicity that ensued in the next two decades. By 1940, the geneticist James Neel, studying *Drosophila*, wondered in a letter to his supervisor Curt Stern whether practitioners “ever thought of the gene as anything except a something that you push around with X-rays” (cited in Campos, 2015). Years of repeatedly using the X-ray machine to manipulate the hereditary material of various organisms thus made Muller’s conclusion that X-rays transmuted genes as obvious and habitual to the researchers who worked with the tube as the machine itself. If one questioned how they could know for certain that they were indeed manipulating genes, they would probably reply in Ian Hacking’s (1983) fashion: “So far as I'm concerned, if you can spray them then they are real.”^14^

Within this new research tradition, for which Muller’s interpretation of X-ray mutagenicity represented sedimented knowledge, the Roentgen machine transformed from something researchers worked on to something they worked with*.* New experiments were designed, relying on the X-ray machine as a ready-made resource for producing mutation, without having to maintain the *ClB* and SLIG stocks or reproduce the crosses Muller had performed with the mutant flies. The technologies and techniques required to first arrive at Muller’s interpretation of X-ray mutagenicity were thus retroactively bracketed as unremarkable steps toward achieving a general description of a natural phenomenon. Inasmuch as it came to be applied routinely, together with the machine in which it had sedimented, Muller’s concept of X-ray mutagenicity hence became what Husserl (1970a) called *discovery-concealment*: a novel conception of nature that conceals the historical process leading to its creation, insofar as it sediments as a presumed basis for subsequent research.

As with any technology, the particular concept of X-ray mutagenicity, which had sedimented in the tube, influenced how scientists perceived new scientific objects that they manipulated with the X-ray machine. Consider one experiment where X-rays acted as a ready-made technology: George Beadle and Edward Tatum’s research on physiological genetics, conducted at Stanford University. With the Roentgen tube modified by Clark and Brown, Beadle and Tatum irradiated a different organism, the red bread mold (*Neurospora crassa*), to show that specific genes control specific biochemical reactions (Beadle & Tatum, 1945). The gist of their experiment was to generate mutations in *Neurospora* and then verify which metabolic processes the offspring could still perform by placing the irradiated strains on chemically defined media, consisting of known nutrients. The minimal medium contained a combination of substances on which a strain could survive only if it was capable of executing all biochemical reactions occurring in “healthy” *Neurospora*. Supplements were subsequently added to the minimal culture one by one to identify the precise substance the mutants needed to survive. It would thereby be possible to determine the individual stages of metabolic processes inhibited by each mutation (Beadle & Tatum, 1941; Creager, 2004; Kay, 1989; Kohler, 1991). In the experimental run, which provided decisive evidence for their one gene-one enzyme hypothesis, Beadle and Tatum employed the X-ray tube as a technology for spawning mutations. The machine imposed on their research object a technological parameter that was fundamental for their claim. As they acknowledged themselves, their entire experiment was “based on the *assumption that X-ray treatment will induce mutations in genes* concerned with the control of known specific chemical reactions” (Beadle & Tatum, 1941, emphasis added). In other words, they took for granted Muller’s conclusion that X-rays primarily modified *genes*, not other aspects of inheritance. Intriguingly, they declared this assumption as an anonymised statement of natural fact, without even quoting Muller or justifying it any further. Beadle and Tatum thus applied the concept of X-rays as transmuters of genes routinely, along with the Roentgen machine. Due to the concept of X-ray mutagenicity sedimented in the X-ray tube, the manifold genetic factors that might participate in the regulation of metabolism were reduced to genes alone. The “one gene” side of the one gene-one enzyme hypothesis was presumed established by sheer virtue of using the X-ray tube.

Strikingly, Beadle and Tatum made this assumption despite new findings running against Muller’s initial belief that X-rays caused only gene mutation. Indeed, Muller would personally revisit his earlier view of X-rays as artificial transmuters of genes. Through cytological analysis of *Drosophila* mutants’ chromosomes, he observed that X-rays provoked not merely gene or “point” mutations, but also translocations of entire chromosomal segments (Muller & Painter, 1929; Muller, 1928c; Schwartz, 2008; Stadler, 1932). Another major issue was Muller’s conviction that X-ray-induced mutation was representative of all spontaneously arising mutation, a position he would defend repeatedly (1928b, 1954). Some researchers challenged his perspective, especially Lewis Stadler, experimenting with plants. Stadler (1932) insisted that X-rays can only cause types of mutation stemming from chromosomal breakage—deletions, chromosomal interchange, loss—but are incapable of generating new genes. Anticipating such objections, Muller conducted additional experiments in which he tried using X-rays to reverse known spontaneous mutations back to the normal-type. Together with two co-workers in Texas, he managed to reverse some mutants, like the forked bristles and bar-eye *Drosophila*. He presented this as proof that X-rays can also induce “progressive” forms of change, not just “break-down processes” (Hanson, 1928; Muller, 1928b, 1929; Patterson & Muller, 1930). However, since he failed with many other mutants, it remained possible that the successfully reversed mutants might have been duplications.^15^ The effect of X-rays could therefore still be interpreted as a deletion of duplicated sequences, rather than an authentic creation of genes. In the absence of methods for analysing the chemical nature of the induced changes, it remained an experimentally undecided dilemma whether X-ray-induced mutations were representative of all mutation and, consequently, precisely what kind of abnormalities were actually being engendered with X-ray technology in other fields.

Although Stadler had been right from the perspective of today’s knowledge, his rebuttals did not prevent the X-ray tube from becoming a genetic technology. Beadle and Tatum still assumed it as a device for incising individual genes despite results indicating that X-ray-induced mutation might not be limited to the level of the gene and that X-rays possibly provoked merely destructive chromosomal changes. In their articles no reference is given to these new findings of radiation genetics and it is probable that they were not aware of them at all. While it is true that Stadler merely raised doubts against Muller’s interpretation of X-ray mutagenicity, without conclusively proving it wrong, his findings potentially affected the foundational assumption of Beadle and Tatum’s work: that X-rays manipulated individual genes. The case therefore shows how taken for granted past knowledge can become through repeated practical and collective use of a material, i.e., through technological sedimentation.

Conversely, it also demonstrates that the sedimentation of Muller’s conclusions did not forestall controversy in other scientific fields where radiation mutagenicity was still actively explored. A separate tradition of researchers, like Stadler and—to a lesser extent—Muller in his later work, did not fully accept Muller’s initial interpretation of X-rays as artificial transmuters of genes and proceeded to explore the genetic effects of X-rays as an object of research, rather than use the tube as a ready-made technology for producing mutants. Among these scientists, Muller’s concept of X-ray mutagenicity did not sediment. X-ray mutagenicity hence existed in parallel as a sedimented concept and object of research, depending on the given research tradition. Scientists acting within separate traditions in the same period may therefore consider the same material thing in opposite ways: as a technology and as an object of inquiry.

## Conclusion

The aim of the article has been to offer a new understanding of technology in scientific research by viewing it as the outcome of a particular mode of sedimentation of scientific thought. This technological sedimentation can be defined as the socialisation and routinisation of past scientific knowledge through the practical use of materials for manipulating investigated phenomena in experimental situations by a community of researchers. Every element of the definition is equally important for technological sedimentation to occur and consequently for an object to act as a technology in scientific research. First, *past knowledge associated with the material*. An example of such received knowledge in Muller’s 1926–27 experiments was that the fly stocks embodied mutant genes, as well as the knowledge about the genetic properties of the *ClB* and SLIG stocks. For Beadle, Tatum and numerous other geneticists who employed the X-ray machine, the most relevant piece of past knowledge was Muller’s interpretation that X-rays caused gene mutations, not other forms of genetic aberration, and that these genic changes mirrored naturally occurring mutation. Second, *routine collective practical use*. Technological sedimentation occurs when a number of practitioners adopt a machine, model organism or other equipment in their everyday work. As they use an organism or device in concrete experimental situations, practitioners come to accept and apply the concepts associated with this material as routinely as the material itself. Third, *an appropriate medium*. Technological sedimentation is unique due to the physical shape of its medium, which can be employed and shared differently than linguistic forms or visual representations. On the one hand, the particular material properties of technologies allow scientists to use them to physically manipulate new investigated phenomena. On the other hand, practitioners often encounter a standardised research material, like the X-ray tube, as a piece of mundane equipment in their workplace—as a thing that is already there, available for use. To work with it, they merely have to learn how to operate it, without delving into the history or ongoing debates outside their research tradition about what this material precisely does or how this knowledge had been gained. The material medium of technologies hence allows researchers to easily use them without deliberating on the concepts sedimented in them.

Due to sedimentation, interventions with technologies are not neutral. Past scientific knowledge sedimented in technologies affects how scientists perceive new phenomena which they manipulate with them. Because of the concepts sedimented in them, technologies restrict intricate natural phenomena to a set of isolated features or, as I called them, *technological parameters*. Past genetic knowledge sedimented in Muller’s fruit flies, for instance, prompted him to reduce the manifold genetic effects of X-rays to a research object defined by four parameters: recessive X-linked lethal gene mutation. In turn, Muller’s concept of X-ray mutagenicity, sedimented in the X-ray machine, led Beadle and Tatum to limit possible factors that may regulate metabolism to genes. In each of these cases, the knowledge sedimented in the chosen technology brought out certain select aspects of the studied natural phenomenon and thus confined it to a pliable research object.

I proposed that certain other elements involved in the production and spread of scientific knowledge, like images, diagrams, popular science media, rhetoric used in reporting experiments, etc., may be seen as *strategies of sedimentation*. These strategies serve to secure the assent of other scientists to new scientific results and thus establish a community within which newly produced knowledge may sediment.^16^ The detailed descriptions of experiments, tables of data and visual representations of crosses that Muller presented at the fifth International Congress of Genetics (1928b), along with the textbooks and newspaper articles proclaiming his discovery of artificial gene transmutation are examples of “literary” strategies, aimed at persuading other scientists, students and the general public to accept Muller’s claim that X-rays can transform genes. Furthermore, at least three “social” strategies can be recognised in Muller’s case, which precipitate technological sedimentation: (1) standardisation of materials; (2) metrology, reflected in the relatively established units for measuring doses of Roentgen radiation; (3) heuristics, training and discipline which codify and homogenize the techniques required to properly use a technology. Together, these strategies allow scientists located in spatially distant research facilities to assume that they are working with materials sufficiently similar, in a manner comparable enough to not cause meaningful discrepancies between their results. Standardisation, metrology and training therefore allow a piece of equipment to travel more inconspicuously and extend the community of scientists that can use it, thus accelerating the process of technological sedimentation. The X-ray machine was, for instance, such a powerful medium for the sedimentation of Muller’s concept of X-ray mutagenicity because of the commercial accessibility of catalogue models of X-ray machines as well as the widespread employment and cooperation of trained radiologists in genetic experiments with X-ray tubes in the US in the late 1920s.^17^

Although in Muller’s example these strategies managed to largely overcome arguments mobilised against his broad interpretation of X-ray mutagenicity, the case also shows that sedimentation does not imply universal acceptance, even in instances of highest scientific success. Concepts usually sediment among a bounded community of scientists while remaining objects of research and dispute for others. Sedimentation is *local*, transpiring within particular research traditions, and *provisional*, contingent on new findings, reshaped alliances and scientific communities. One major reason for the provisional character of sedimentation is the underdetermination of technological parameters. Future investigation may always cast doubt on the selection of parameters through which technologies had framed research objects, or on the extrapolations scientists have made from these parameters in constructing more general conceptual abstractions. In Muller’s case, the questionable parameter was reducing the genetic effects of X-rays to gene mutation, whereas his most controversial generalisation was that X-ray-induced mutation is equivalent to naturally occurring mutation. Sedimentation therefore shapes revisable traditions. The concepts layered in technologies are not akin to Lakatos’ (1978) irrefutable hard cores; they are susceptible to being re-evaluated, altered and sometimes supplanted altogether.

## Footnotes

1 Their account was for instance adopted by Kohler (1994) in his study of the fruit fly as a technology in classical genetics.

2 Despite shifts in Latour’s position, he retains this observation in his later discussions about technical objects (1994, 1999).

3 See Shapin (1988), Hacking (1992) and Baird (2004) for consonant criticisms of Latour’s fixation on literary production.

4 The extension of sedimentation to material culture finds support in Husserl’s *Crisis of European Sciences* (Husserl, 1970a), where he briefly hints that sedimentation can also occur in cultural artefacts (*Kulturobjekte*), like tongs and drills. Steinle (2010) advocates a similar understanding of sedimentation.

5 Technological parameters are further discussed in Sect. 4. Galison (1987 p. 251) introduces a related notion, *technological presuppositions*, to highlight that “machines are not neutral”, but does not develop it further.

6 Cf. Latour (1994) who similarly stresses that technologies are not neutral intermediaries. Technical mediation is rather a translation, which shifts the meaning of past science congealed in technologies.

7 The only exception I found is the 1915 collective monograph (Morgan et al., 1915 p. 3, p. 208). Even here the term factor is defined only passingly, as a “something” that affects the organism’s observable characters.

8 My interpretation merely describes a predominant tendency among the Morgan group. It does not imply that they were unaware of other genetic aberrations than gene mutations or that they did not study them in certain experiments (e.g., Bridges’ 1913 article on non-disjunction).

9 In this respect, sedimentation expands not only on Ludwik Fleck’s points about the mutual relationship between *thought collective* and *thought style*, but also on the role of tradition in what sociologists of scientific knowledge call *meaning finitism* (Fleck, 1986[1947]; Barnes et al., 1996). See also Latour’s (1999) remark that concepts, i.e. the contents of science, are what holds collectives together and, in turn, acquire scientific status by reason of belonging to these associations.

10 Muller also used a strain with balanced lethals inserted on the second chromosome for some of his temperature experiments, but abandoned it in the X-ray runs (Muller, 1928a, 1928b), and an attached-X culture of females in his third X-ray experiment, which mostly served to verify results from the first two series (Muller, 1928b). The argument I make about *ClB* and SLIG applies to these stocks as well.

11 Some secondary sources conflate the SLIG and sex-ratio tests (e.g., Carlson, 1981). To avoid ambiguity, I stick to Muller’s own expression, although it deviates from standard terminology in genetics.

12 A valuable exception is Campos (2015).

13 Whenever Muller’s concept/interpretation of X-ray mutagenicity is mentioned in the article, this refers to his interpretation of X-rays as artificial transmuters of genes, as described in Sect. 4, not to the qualifications he made in his later work. It was this initial, broad interpretation that sedimented in the X-ray machine through its adoption by other geneticists.

14 In his famous proposal that manipulability can be taken as a criterion for the existence of scientific entities, Hacking overlooks that the mere possibility of manipulating something does not determine how that something should be perceived (Arabatzis, 2006). What made it obvious to geneticists that the something they were manipulating was a gene, was not manipulation itself, but Muller’s concept of X-ray mutagenicity sedimented in the X-ray machine. This loss of distinction between a technology’s physical interaction with the observed phenomenon and scientists’ interpretation of what the technology is doing, is a result of successful technological sedimentation.

15 And most likely were, at least according to current research (Ishimaru et al.,^1995^; Wolfner & Miller, 2016).

16 Strategies of sedimentation largely overlap with what Shapin and Schaffer (2011[1985]) refer to as social and literary technologies. To an extent this is in accordance with Shapin and Schaffer’s own interpretation since they sometimes refer to technologies as “strategies for knowledge-production” (2011 [1985] p. 104, also pp. 76–77).

17 The list of strategies in the paragraph is not meant to be exhaustive. More could be said, for instance, about calibration (Collins, 1985) as a social strategy of sedimentation.
